# Exploratory Stage-Resolved Acoustic Emission Characterization During Tensile Testing of Furnace-Exposed S235 Structural Steel †

**DOI:** 10.3390/ma19214495

**Published:** 2026-10-26

**Authors:** Anna Adamczak-Bugno

**Affiliations:** Faculty of Civil Engineering and Architecture, Kielce University of Technology, Av. 1000-An. of Polish State 7, 25-314 Kielce, Poland; aadamczak@tu.kielce.pl

**Keywords:** acoustic emission, S235 structural steel, thermal exposure, tensile deformation, fracture behaviour, mechanical properties, material characterization, absolute energy

## Abstract

**Highlights:**

- Individual AE hits were referenced to four specimen-specific tensile-deformation stages.
- Median AE amplitude was generally highest before macroscopic yielding.
- Duration, rise time, and absolute energy showed greater variability than amplitude.
- Temporal and energetic AE descriptors varied among the available furnace-exposure records and deformation stages.
- Separate channel analysis demonstrated measurement-path dependence of the descriptor levels.

**Abstract:**

Thermal exposure changes the residual mechanical response of structural steel and may modify acoustic emission (AE) recorded during subsequent loading. This exploratory study examined individual AE hits recorded during tensile testing of an S235 steel product after exposure at nominal furnace temperatures of 700, 900, or 1100 °C for 40, 80, or 120 min, followed by still-air cooling. The mechanical programme comprised 30 specimens across ten conditions, whereas archived AE records suitable for stage-resolved analysis were available for 20 specimens. Hits were assigned to specimen-specific pre-yield, yielding, plastic-deformation, and post-ultimate-tensile-strength stages. Amplitude, duration, rise time, and absolute energy were summarized using the specimen as the unit of analysis. The largest residual-strength reductions occurred after the 1100 °C exposures. In the available AE records, median amplitude was generally highest during Stage I, which included both early material-related activity and possible contributions from load introduction and specimen–grip interaction. Temporal and energetic descriptors showed greater inter-specimen and channel-dependent variation than amplitude. The results provide an exploratory description of stage-dependent AE characteristics under the investigated nominal furnace-exposure conditions; they do not establish statistically confirmed effects among all ten conditions.

## 1. Introduction

Structural steel is widely used in buildings and infrastructure because of its favourable combination of strength, ductility, manufacturability, and structural efficiency. A thematically related conference abstract reported a separate investigation of thermally exposed S235 steel using acoustic emission, numerical analysis, and metallographic observations [1]. During fire exposure, however, increasing temperature reduces stiffness and load-bearing capacity and may lead to excessive deformation, instability, or structural failure [2,3,4]. After cooling, part of the mechanical capacity may be recovered, but sufficiently severe thermal exposure can produce persistent changes in residual yield strength, tensile strength, ductility, residual-stress state, and subsequent deformation behaviour [5,6,7,8]. The residual material condition depends not only on the maximum temperature but also on exposure duration and the cooling path [9,10,11].

Post-fire evaluation of structural steel is commonly based on visual inspection, geometrical measurements, hardness testing, microstructural observations, and mechanical characterization [12,13,14]. Uniaxial tensile testing remains one of the principal methods for determining residual yield strength, ultimate tensile strength, and deformation capacity [5,7,9]. These parameters quantify the macroscopic consequences of thermal exposure but do not directly describe localized dynamic processes activated during subsequent loading. Materials with similar residual strength parameters may therefore exhibit different deformation histories and different patterns of damage development.

Acoustic emission (AE) monitoring provides complementary information because it records transient elastic waves generated by dynamic and irreversible processes occurring in a loaded material [15,16,17]. In metals, detectable AE activity may accompany dislocation motion, discontinuous yielding, localized plastic deformation, crack initiation and propagation, and final fracture [18,19,20]. The relative contribution of these sources changes with material state and deformation stage, while the detected waveform is additionally affected by propagation and the measurement chain. AE has therefore been used to investigate tensile deformation, plastic instabilities, fatigue damage, crack growth, and fracture in metallic materials, including structural, pipeline, and prestressing steels [21,22,23].

Interpretation of an AE record depends strongly on the selected descriptor. Cumulative counts or cumulative hit numbers describe the overall development of detectable activity, but they integrate signals generated by potentially different processes and do not characterize the individual hits contributing to the accumulated response [17,24,25]. Similar final cumulative activity may therefore be produced by records that differ substantially in signal amplitude, duration, rise time, energy, frequency content, or in the mechanical stages during which the signals occur.

Amplitude represents the maximum recorded level of an AE waveform on a logarithmic decibel scale. Duration describes the interval between the first and final threshold crossings, whereas rise time is the interval between the first threshold crossing and maximum signal amplitude [17,26]. Absolute energy characterizes the time-integrated signal intensity and can differentiate hits with comparable amplitudes but different waveform duration and content [17,27]. These descriptors depend on the source process, propagation path, sensor response, coupling, acquisition threshold, and hit-definition parameters [28,29,30]. They should therefore be interpreted as configuration-dependent comparative quantities rather than intrinsic material properties.

Previous investigations have demonstrated that AE signal analysis can support the characterization of damage development in different construction materials. Time–frequency analysis of AE signals recorded during tensile testing of glass-fibre-reinforced polymer composites enabled changes associated with the progression of destructive processes to be identified [31]. In fibre–cement composites, AE descriptors combined with signal classification and wavelet analysis were used to differentiate materials according to reinforcement condition and mechanical performance [32]. AE-based assessment was subsequently incorporated into a broader monitoring concept developed for fibre–cement panels used in ventilated façade systems [33]. Nevertheless, these studies concerned composite materials and did not address the stage-resolved evolution of scalar AE hit characteristics during tensile deformation of thermally exposed structural steel.

Mechanically resolved analysis is particularly important because the dominant deformation processes change during tensile loading. In low-carbon structural steel, the pre-yield interval, discontinuous-yielding region, subsequent strain hardening, and post-UTS localization constitute mechanically distinct stages [18,19,34]. Pooling AE hits from the entire test can conceal differences between these intervals. Assignment of individual hits to specimen-specific mechanical stages makes it possible to determine whether thermal exposure changes the acoustic response uniformly or selectively modifies particular periods of deformation.

The objective of this exploratory study is to characterize how amplitude, absolute energy, duration, and rise time vary across specimen-specific stages of tensile deformation in the available AE records from furnace-exposed S235 steel. The analysis examines whether stage-resolved patterns differ among the investigated nominal temperature–duration groups and whether temporal and energy-related descriptors provide information not apparent from amplitude alone. The study does not test a population-level causal effect of thermal history. Its methodological contribution lies in combining specimen-specific mechanical segmentation with hit-level AE characterization while retaining the two acquisition channels as separate measurement paths.

## 2. Materials and Methods

### 2.1. Material and Specimen Preparation

The investigation was conducted on S235 low-carbon structural steel in the as-received condition and after exposure under nominal high-temperature furnace conditions [35]. The material was identified as S235 on the basis of the available material documentation. Independent grade verification and detailed chemical-composition data were not available. The findings are therefore specific to the investigated product and should not be generalized to all heats and products classified as S235 or interpreted in terms of particular phase transformations.

Flat tensile specimens were cut from hot-rolled steel plate parallel to the rolling direction. The specimens had a symmetrically tapered reduced section whose width decreased from approximately 10 mm near the transition radii to 6 mm at the centre of the gauge section. The initial thickness was approximately 5 mm, and the extensometer gauge length was 25 mm. Dimensions were measured individually with a precision calliper and micrometre having a resolution of 0.01 mm. Engineering stress was calculated using the measured cross-sectional area at the minimum-width section.

### 2.2. Thermal Exposure

Specimens were exposed to nominal furnace temperatures of 700, 900, and 1100 °C for 40, 80, or 120 min. Specimens maintained at room temperature without heat treatment formed the reference condition. The complete mechanical programme comprised 30 specimens, with three specimens assigned to each of the ten conditions.

Thermal exposure was performed in a chamber furnace with a temperature-control accuracy of approximately ±5 °C. The specimens were placed in the furnace at ambient temperature and heated together with the furnace. Furnace temperature was monitored using a type-K thermocouple located in the central region of the chamber. Exposure time was measured from the moment at which the furnace reached the prescribed nominal temperature. After the exposure period, the specimens were removed and cooled in still laboratory air.

The reported temperatures represent nominal furnace temperatures rather than directly measured specimen temperatures because no thermocouples were attached to the specimens. Complete specimen-specific heating and cooling curves, heating rates, cooling rates, time to specimen thermal equilibrium, furnace spatial-uniformity measurements, and removal-delay records were not available. The furnace operated in laboratory air. After cooling, oxide scale was removed mechanically and the specimen dimensions used for stress calculations were measured again. Consequently, the nominal furnace-exposure groups should not be interpreted as fully verified specimen thermal histories.

### 2.3. Uniaxial Tensile Testing

Uniaxial tensile tests were conducted at ambient temperature using a Zwick/Roell universal testing machine (ZwickRoell GmbH & Co. KG, Ulm, Germany) equipped with a class 1 load-measurement system in accordance with PN-EN ISO 6892-1:2020-05 [36]. The tests were performed under displacement control at a crosshead displacement rate of 5 mm/min. This value represents the machine-control parameter and should not be interpreted as a directly measured constant strain rate within the gauge section. Gauge-section strain was measured using a mechanical extensometer with an initial gauge length of 25 mm and displacement resolution of approximately 1–2 µm. Tests were performed at 21 ± 1 °C and a relative humidity of approximately 40–50%.

Engineering stress was calculated as σ = F/A_0_, where F is the instantaneous tensile force and A_0_ is the individually measured initial cross-sectional area after thermal exposure, cooling, and scale removal. Engineering strain was calculated as ε = ΔL/L_0_, where ΔL is the extensometer displacement and L_0_ = 25 mm.

### 2.4. Acoustic Emission Monitoring

AE was recorded continuously using an Express-8 eight-channel acoustic-emission acquisition system controlled by AEwin software (MISTRAS Group, Inc., Princeton Junction, NJ, USA). Two Vallen VS75-SIC-40dB sensors (Vallen Systeme GmbH, Wolfratshausen, Germany) with integrated 40 dB preamplifiers and a nominal resonant frequency of 75 kHz were used. The sensors were mounted on opposite sides of the specimen and mechanically secured to maintain contact during loading. Technical silicone was used as the coupling medium. Before each test, sensor coupling and signal transmission were verified using reproducible Hsu–Nielsen pencil-lead breaks [37].

The acquisition threshold was 40 dB. This fixed threshold was selected during preliminary system checks as an operational level intended to suppress observable low-level background and testing-system noise while retaining mechanically induced activity. A separate quantitative background-noise distribution was not archived. The same threshold, filter, sampling rate, sensor type, coupling procedure, and hit-definition settings were applied in all tests. The sampling frequency was 10 MHz, and the band-pass filter was 20–400 kHz. The peak definition time, hit definition time, and hit lockout time were 200, 800, and 1000 µs, respectively. The two sensors were connected to independent acquisition channels and processed using identical settings. Signals from the channels were not combined because the same physical source could be detected by both sensors. Channel 1 was used as the common primary display channel, whereas Channel 2 was evaluated separately as a measurement-path sensitivity assessment. The second channel was not treated as an independent experimental replicate.

**Figure 1:**
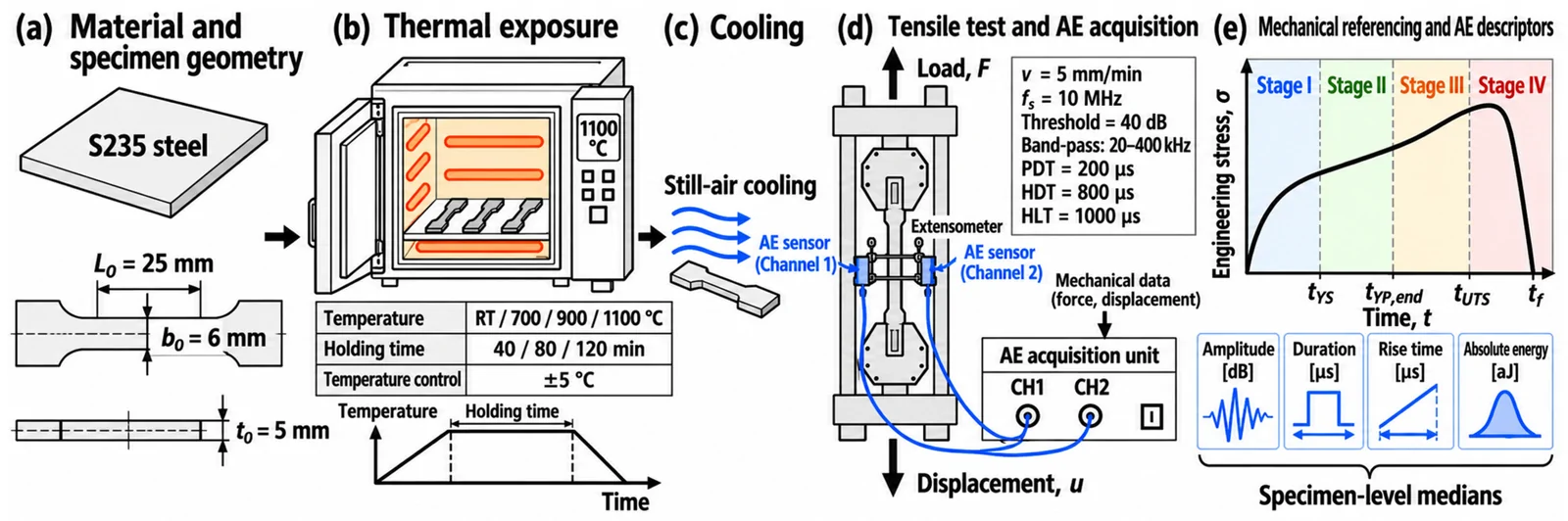
Experimental workflow used for the stage-resolved characterization of acoustic emission during tensile deformation of thermally exposed S235 structural steel: (**a**) investigated material and tensile-specimen geometry; (**b**) thermal-exposure conditions; (**c**) cooling in still laboratory air; (**d**) tensile testing with two-channel AE monitoring and the principal acquisition settings; and (**e**) mechanical referencing of individual AE hits to four deformation stages and the evaluated signal descriptors. AE, acoustic emission.

The complete experimental workflow, including specimen preparation, thermal exposure, air cooling, tensile testing, two-channel AE acquisition, mechanical stage referencing, and extraction of the analyzed AE descriptors, is summarized in Figure 1.

### 2.5. Synchronization and Definition of Deformation Stages

Mechanical and AE datasets were exported and placed on a common time axis. A constant time offset was applied by aligning the onset of AE acquisition with the beginning of the increase in the force–time record. No local stretching, nonlinear time transformation, or stage-dependent correction was applied. Characteristic mechanical times were determined separately for each specimen.

Four tensile-deformation stages were defined: Stage I, pre-yield (*t* < *t*_YS_); Stage II, yielding (*t*_YS_ ≤ *t* < *t*_YP,end_); Stage III, plastic deformation and strain hardening (*t*_YP,end_ ≤ *t* < *t*_UTS_); and Stage IV, post-UTS localization and fracture (*t*_UTS_ ≤ *t* ≤ *t*_f_). Here, *t*_YS_ denotes the time of the upper yield point initiating the yield drop, *t*_UTS_ is the time of maximum engineering stress, and *t*_f_ is the time of final fracture. The end of the yield plateau was identified individually by visual inspection of the corresponding stress–time curve as the onset of a sustained stress increase associated with strain hardening. The selected boundary was additionally checked against the stress–strain response. Because the extent and shape of the yield plateau differed among specimens, no single universal time interval or slope threshold was imposed. When a distinct upper yield point and yield plateau could not be resolved, Stage II was not assigned. This occurred for specimen S235_1100_80_1; its data from the remaining identifiable stages were retained. The mechanical boundaries and the resulting four-stage division are illustrated schematically in Figure 1e.

### 2.6. AE Descriptors and Statistical Summarization

The investigated hit-level descriptors were amplitude [dB], duration [µs], rise time [µs], and absolute energy [aJ]. Absolute energy was exported directly from the AE acquisition software as the system-defined time-integrated signal quantity for each hit. It is influenced by waveform voltage, hit duration, acquisition electronics, sensor response, coupling, and propagation and is therefore not interpreted as independently calibrated source energy. Because absolute energy was strongly right-skewed, it was transformed as log_10_(*E*_abs_ + 1), where *E*_abs_ is expressed in aJ. For orientation, transformed values of 2, 3, and 4 correspond to approximately 99, 999, and 9999 aJ, respectively. Duration and rise time were displayed on logarithmic axes where appropriate, but specimen-level summaries were calculated in the original units.

Stage-resolved hit-level analyses were performed for 20 of the 30 mechanically tested specimens for which synchronized mechanical and AE records were available. The remaining ten specimens were included in the mechanical testing programme, but no archived AE records suitable for stage-resolved reanalysis were available for these tests. Because the unavailable AE records could not be retrospectively evaluated, it was not possible to determine whether their absence was random with respect to thermal condition or mechanical response. These specimens were therefore retained in the mechanical summary but were not included in the stage-resolved AE analysis. The AE results should consequently be interpreted as an exploratory analysis of the available records rather than as a balanced comparison of all three specimens representing each exposure condition.

Available specimen–channel–stage combinations were analyzed separately; an incomplete recording did not invalidate data from the other available stages of the same specimen. Of the 160 theoretically possible combinations for the 20 specimens with AE data, 156 were available for analysis. The unavailable combinations comprised Stage IV for both channels of specimen S235_0_2, whose AE recording ended before UTS, and Stage II for both channels of specimen S235_1100_80_1, for which a distinct upper yield point and yield plateau could not be resolved. Missing combinations were excluded from the corresponding summaries and were not interpreted as zero AE activity. Detailed accounting of all mechanically tested specimens, including AE-record availability, usable channels and stages, and reasons for incomplete analysis where these could be established from the archived records, is provided in Supplementary Table S1.

Across both channels, 435,172 hits were analyzed: 307,132 for Channel 1 and 128,040 for Channel 2. The specimen was treated as the unit of analysis; individual AE hits were not considered independent material replicates. For every record, channel, and deformation stage, the median and interquartile range of each descriptor were calculated from all available valid hits. Condition-level values were represented by the median and minimum-maximum range of the available specimen-level medians. The analysis was descriptive and exploratory, and no formal significance testing or universal classification thresholds were applied. Hit counts for each specimen, channel, and stage are provided in Supplementary Table S2.

For amplitude–duration visualization only, equal-weight chronological samples containing no more than 120 recorded hits per specimen, channel, and stage were used. When a stratum contained *N* > 120 hits, 120 approximately equally spaced chronological indices were selected from the first to the last hit; this is equivalent to sampling at an interval of approximately *k* = (*N* − 1)/119 while retaining the chronological coverage of the stage. When *N* ≤ 120, every valid hit was retained. The subsampling procedure was used exclusively for visualization. All available valid hits were retained when calculating specimen-level medians and interquartile ranges. No synthetic observations were generated.

### 2.7. AE Validation and Sensitivity Analyses

Three supplementary analyses were performed to evaluate the robustness and measurement-chain dependence of the stage-resolved results. First, the influence of the 40 dB acquisition threshold was assessed by calculating the proportions of all valid hits recorded at exactly 40 dB and at amplitudes not exceeding 41, 42, and 43 dB.

Second, the possible influence of activity associated with initial load introduction was examined for Stage I. The specimen-level Stage I medians were recalculated after excluding hits recorded before the mechanical stress reached 5% and 10% of the specimen-specific UTS. The recalculated amplitude and log_10_(absolute energy + 1) medians were compared with the values obtained from the complete Stage I interval. This analysis was possible for 38 specimen–channel records containing the required synchronized mechanical and AE data.

Third, sensitivity to synchronization uncertainty was evaluated by shifting the mechanical stage boundaries relative to the AE time base by ±0.5 s and ±1.0 s. For each shift, the proportion of hits reassigned to a different deformation stage was calculated. This procedure quantified the extent to which small changes in the synchronization offset could affect stage membership.

Agreement between the two acquisition channels was assessed using paired specimen–stage summaries rather than condition-level medians. For every specimen and stage available in both channels, Channel 1 and Channel 2 values were paired for amplitude, log_10_(absolute energy + 1), duration, and rise time. Channel correspondence was described using Spearman’s rank correlation coefficient, Lin’s concordance correlation coefficient, the mean paired difference calculated as Channel 1 minus Channel 2, and Bland–Altman limits of agreement defined as the mean paired difference ±1.96 standard deviations of the paired differences. These analyses were descriptive and were not used to test thermal-condition effects.

## 3. Results

### 3.1. Residual Mechanical Response

Residual tensile properties varied among the investigated temperature-duration conditions (Table 1). The reference condition had mean lower and upper yield strengths of 324.14 and 351.01 MPa and a mean UTS of 463.06 MPa. The largest strength reductions were observed after the 1100 °C exposures. Differences between nearby condition means, particularly within the 900 °C series, should be interpreted together with the reported standard deviations rather than as evidence of statistical separation.

The upper yield strength was not resolved consistently for the 1100 °C/80 min condition and is therefore not reported. Elongation at fracture was not included because a uniform, complete set of post-fracture elongation measurements was not available for all conditions.

The mean lower yield strength decreased from 324.14 MPa in the reference condition to 266.18, 232.32, and 231.01 MPa after exposure at 1100 °C for 40, 80, and 120 min, respectively. For the investigated specimens, the nominal 1100 °C furnace-exposure groups showed the largest reductions in mean residual strength. These mechanical results provide the reference context for the exploratory interpretation of the available AE records.

### 3.2. Amplitude–Duration Characteristics

The amplitude–duration diagrams showed broad, stage-dependent populations rather than a single compact relationship. Most hits occupied the lower-amplitude region, while progressively smaller populations extended towards higher amplitudes and longer durations. The scatter was particularly wide in the pre-yield and post-UTS stages, indicating that these intervals contained both short, low-amplitude signals and less frequent signals with substantially longer durations.

**Figure 2:**
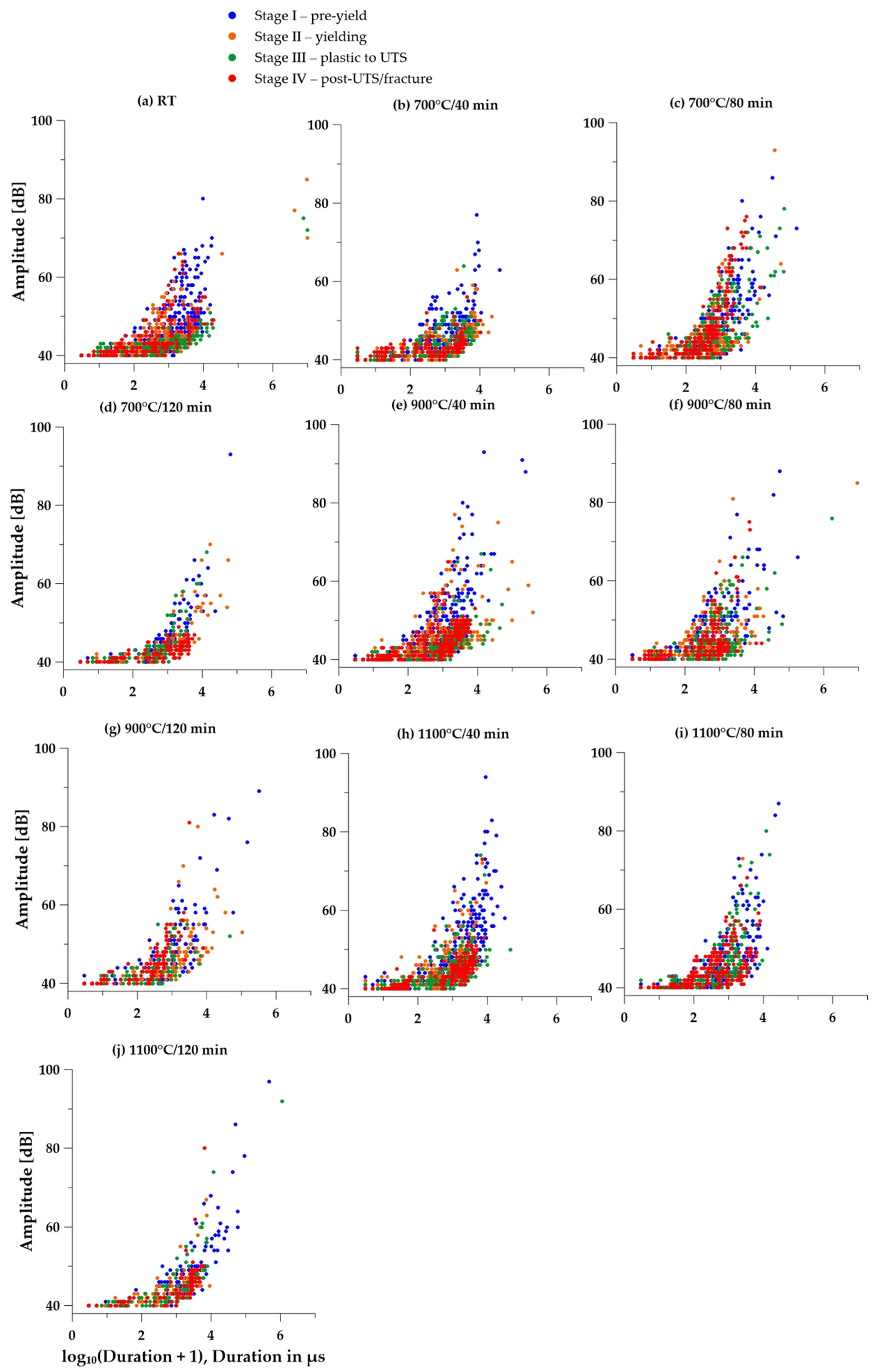
Amplitude versus log_10_(duration [µs] + 1) for systematically sampled Channel 1 AE hits under the ten investigated nominal furnace-exposure conditions: (**a**) RT; (**b**) 700 °C/40 min; (**c**) 700 °C/80 min; (**d**) 700 °C/120 min; (**e**) 900 °C/40 min; (**f**) 900 °C/80 min; (**g**) 900 °C/120 min; (**h**) 1100 °C/40 min; (**i**) 1100 °C/80 min; and (**j**) 1100 °C/120 min. Colours distinguish the four mechanically defined deformation stages. The systematic subsampling was used exclusively for visualization; all available valid hits were retained in the specimen-level calculations. AE, acoustic emission; RT, room temperature.

Thermally exposed conditions did not show a simple monotonic displacement of the complete amplitude–duration population with increasing temperature. Instead, the density and extent of the distributions varied with the combined temperature–duration condition and deformation stage. This observation supports separate analysis of amplitude and temporal descriptors rather than interpretation of amplitude as a surrogate for signal duration. The complete amplitude–duration distributions are presented in Figure 2.

### 3.3. Stage-Resolved Amplitude

Amplitude exhibited the clearest common stage-related tendency among the investigated descriptors. Across the thermal conditions, the Channel 1 condition medians in Stage I ranged from 44 to 47 dB, with a median across conditions of 45 dB. During Stage II, condition medians ranged from 42 to 45 dB, followed by 41–44 dB in Stage III and 42–45 dB in Stage IV. Thus, median amplitude was generally highest before yielding and decreased after the transition into yielding and plastic deformation.

**Figure 3:**
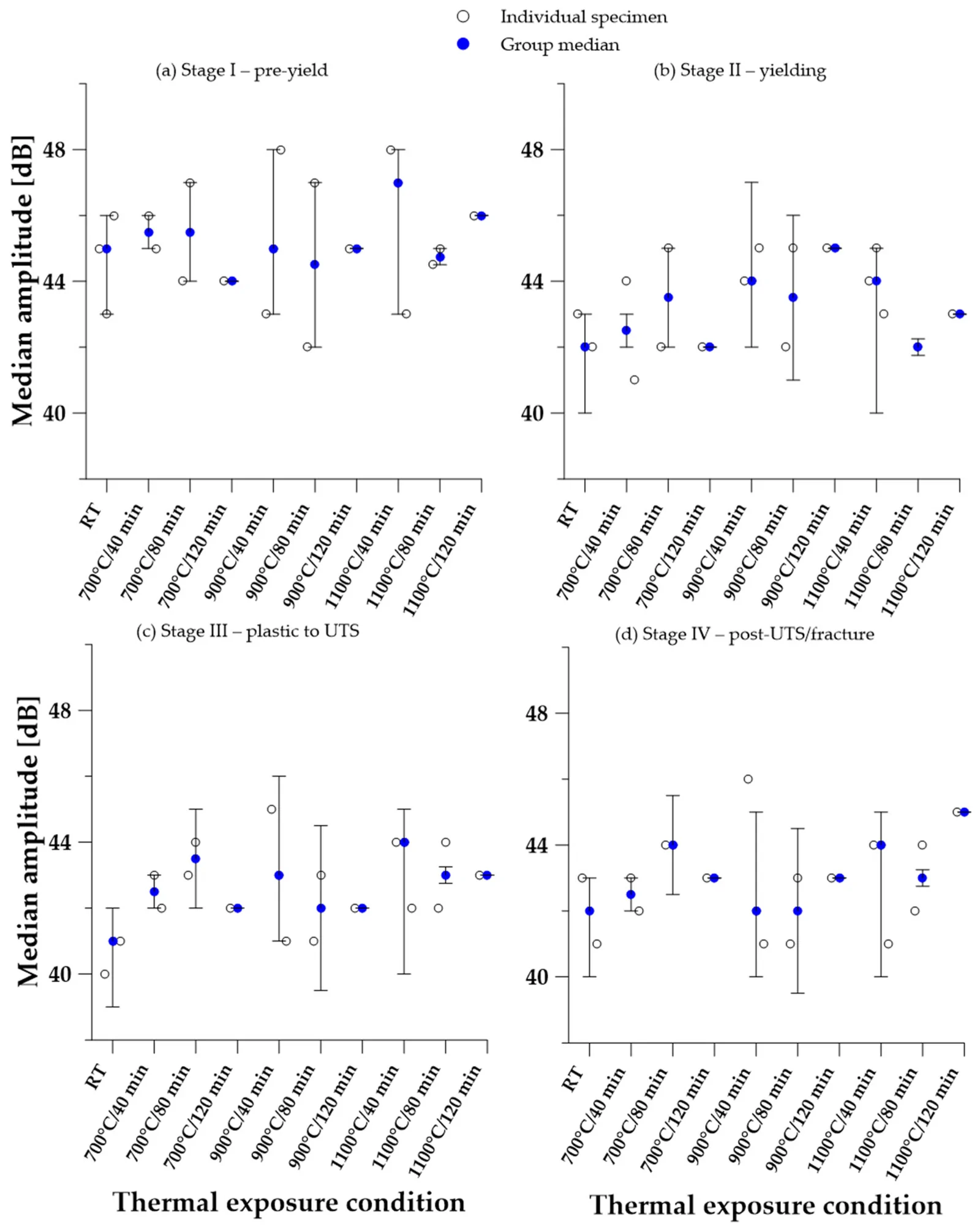
Specimen-level median amplitude recorded by Channel 1 for the investigated nominal furnace-exposure conditions: (**a**) Stage I—pre-yield; (**b**) Stage II—yielding; (**c**) Stage III—plastic deformation to UTS; and (**d**) Stage IV—post-UTS localization and fracture. Open symbols represent individual specimen medians, filled symbols represent condition medians, and vertical bars show the observed minimum–maximum range. Conditions represented by one available specimen are shown without a range and should be interpreted as exploratory observations. UTS, ultimate tensile strength.

The reference condition decreased from a Stage I median of 45 dB to 42 dB in Stage II, 41 dB in Stage III, and 42 dB in Stage IV. Comparable reductions were observed for several thermally exposed conditions. The highest Stage I condition median, 47 dB, occurred after exposure at 1100 °C for 40 min. However, the relatively narrow overall range of the condition medians demonstrates that amplitude alone differentiated the thermal histories only weakly. The specimen- and condition-level amplitude results are presented in Figure 3.

### 3.4. Stage-Resolved Absolute Energy

Absolute energy showed substantially greater condition-dependent variability than amplitude. In Stage I, the Channel 1 condition medians of log_10_(absolute energy + 1) ranged from 2.26 to 3.05. The ranges for Stages II, III, and IV were 1.83–2.65, 1.83–2.82, and 1.97–3.36, respectively. The median of the condition-level values decreased from 2.61 in Stage I to 2.31 in Stage II and remained close to 2.38 and 2.32 in Stages III and IV.

**Figure 4:**
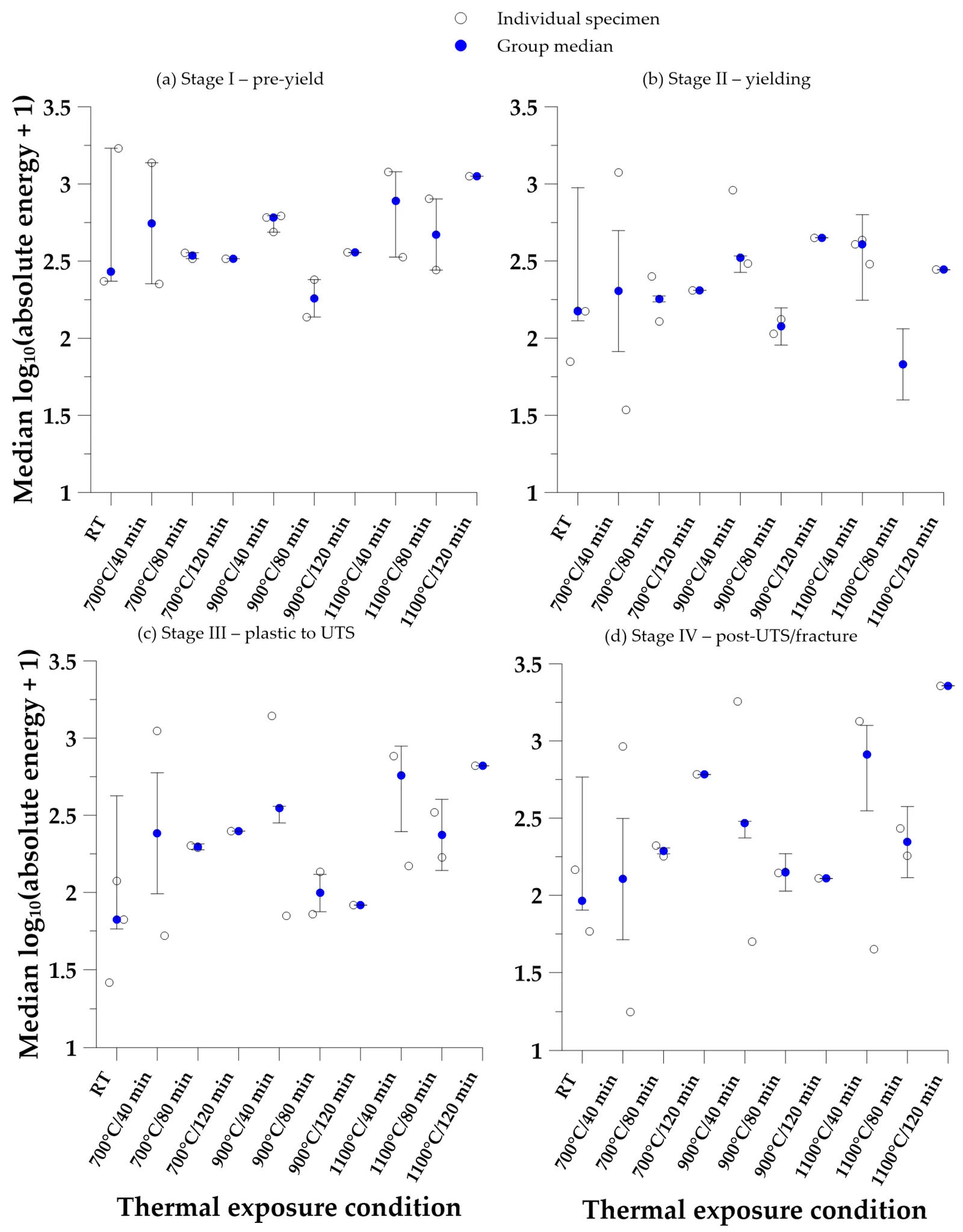
Specimen-level median log_10_(absolute energy + 1) recorded by Channel 1 for the investigated nominal furnace-exposure conditions: (**a**) Stage I—pre-yield; (**b**) Stage II—yielding; (**c**) Stage III—plastic deformation to UTS; and (**d**) Stage IV—post-UTS localization and fracture. Open symbols represent individual specimen medians, filled symbols represent condition medians, and vertical bars show the observed minimum–maximum range. Conditions represented by one available specimen are shown without a range and should be interpreted as exploratory observations.

The reference condition showed a progressive reduction from 2.43 in Stage I to 2.17, 1.83, and 1.97 in Stages II–IV. In contrast, several thermally exposed conditions retained elevated energy during later deformation. The 1100 °C/40 min condition had medians of 2.89, 2.61, 2.76, and 2.91 across Stages I–IV. The highest displayed Stage IV value was observed for the single available 1100 °C/120 min AE record and is therefore reported descriptively. These results indicate that the post-UTS energetic response can differ substantially even when median amplitudes remain within a comparatively narrow interval. The corresponding specimen- and condition-level absolute-energy results are shown in Figure 4.

### 3.5. Stage-Resolved Duration

Duration varied over a much wider relative range than amplitude. Stage I condition medians ranged from 428 to 1841 µs. The corresponding ranges were 131–1152 µs in Stage II, 210–1228 µs in Stage III, and 200–2432 µs in Stage IV. Across conditions, the median condition value decreased from 885 µs in Stage I to 522 µs in Stage II and 473 µs in Stage III, followed by a partial increase to 619 µs in Stage IV.

**Figure 5:**
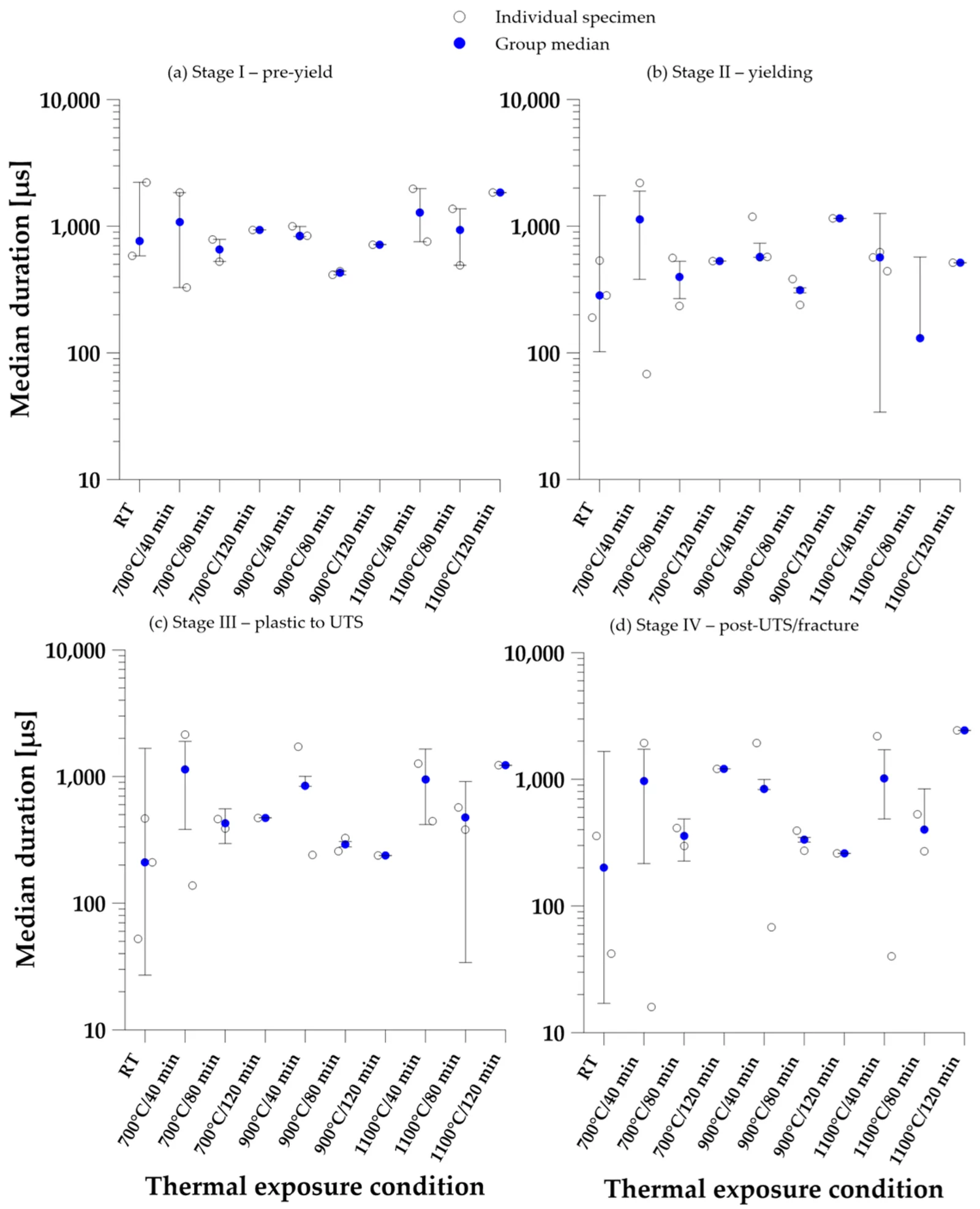
Specimen-level median duration recorded by Channel 1 for the investigated nominal furnace-exposure conditions: (**a**) Stage I—pre-yield; (**b**) Stage II—yielding; (**c**) Stage III—plastic deformation to UTS; and (**d**) Stage IV—post-UTS localization and fracture. Duration is presented on a logarithmic scale. Open symbols represent individual specimen medians, filled symbols represent condition medians, and vertical bars show the observed minimum–maximum range. Conditions represented by one available specimen are shown without a range and should be interpreted as exploratory observations.

For the reference condition, median duration decreased from 766 µs in Stage I to 285 µs in Stage II, 210 µs in Stage III, and 200 µs in Stage IV. Longer signals persisted in several heated conditions. For example, the 1100 °C/40 min condition exhibited medians of 1282, 564, 948, and 1018 µs, whereas the 1100 °C/120 min record exhibited a pronounced Stage IV value. The large minimum–maximum ranges observed for selected conditions demonstrate substantial specimen-to-specimen variability in temporal signal characteristics. The stage-resolved duration results are presented in Figure 5.

### 3.6. Paired Comparison of the Acquisition Channels

**Figure 6:**
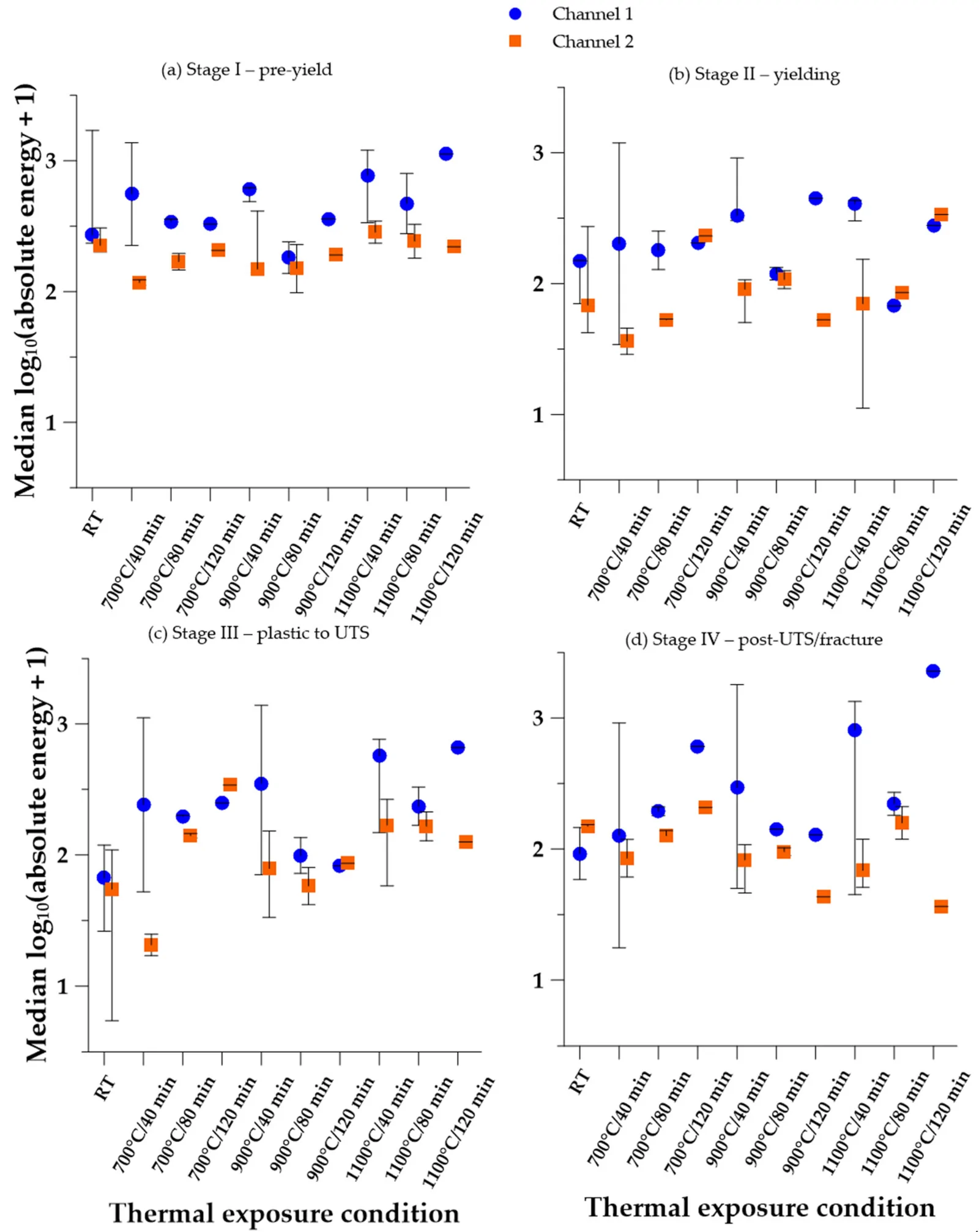
Comparison of specimen-level median log_10_(absolute energy + 1) obtained independently from Channels 1 and 2 across the four mechanically defined deformation stages. Filled symbols represent condition medians, and vertical bars show the observed minimum–maximum range of the available specimen-level medians. Conditions represented by one available specimen are shown without a range. The channels were analyzed separately and were not treated as interchangeable measurements.

Channel agreement was evaluated using 78 paired specimen–stage summaries available simultaneously for Channels 1 and 2. The correspondence depended strongly on the evaluated descriptor (Table 2). Amplitude showed the highest rank correlation, with Spearman’s ρ = 0.434, but its concordance correlation coefficient was only 0.291. The mean paired difference, calculated as Channel 1 minus Channel 2, was −1.673 dB, and the Bland–Altman limits of agreement extended from −6.670 to 3.324 dB. The comparison of Channel 1 and Channel 2 absolute-energy values is presented in Figure 6.

Agreement was weaker for the remaining descriptors. For log_10_(absolute energy + 1), Spearman’s ρ was 0.198 and the concordance correlation coefficient was 0.099. Duration and rise time showed little correspondence between channels, with Spearman’s coefficients of 0.076 and 0.011, respectively, and concordance correlation coefficients close to zero. The wide limits of agreement demonstrate that measurements obtained from the two sensor positions should not be treated as interchangeable.

These results confirm that the two channels represented separate measurement paths rather than independent repetitions of the same scalar measurement. Consequently, the channels were not pooled, and the principal Channel 1 results should be interpreted as configuration-dependent observations. The complete paired specimen–stage values are provided in the Supplementary Materials.

The channel comparison indicates that the main Channel 1 results should be interpreted as configuration-dependent signal characteristics rather than intrinsic source quantities. Channel 2 provides a measurement-path sensitivity check, but it does not reproduce identical absolute values. Full paired condition-stage medians for all four descriptors are reported in Supplementary Table S3.

### 3.7. Stage-Resolved Rise Time

Rise time followed a broadly similar but more variable stage pattern than duration. Stage I condition medians ranged from 20 to 306 µs, whereas Stages II and III ranged from 12 to 305 µs and from 6 to 341 µs, respectively. The widest range occurred in Stage IV, where the condition medians extended from 17 to 956 µs. Across conditions, the median rise time decreased from 138 µs in Stage I to 55 µs in Stage II, increased slightly to 73 µs in Stage III, and rose to 189 µs in Stage IV.

**Figure 7:**
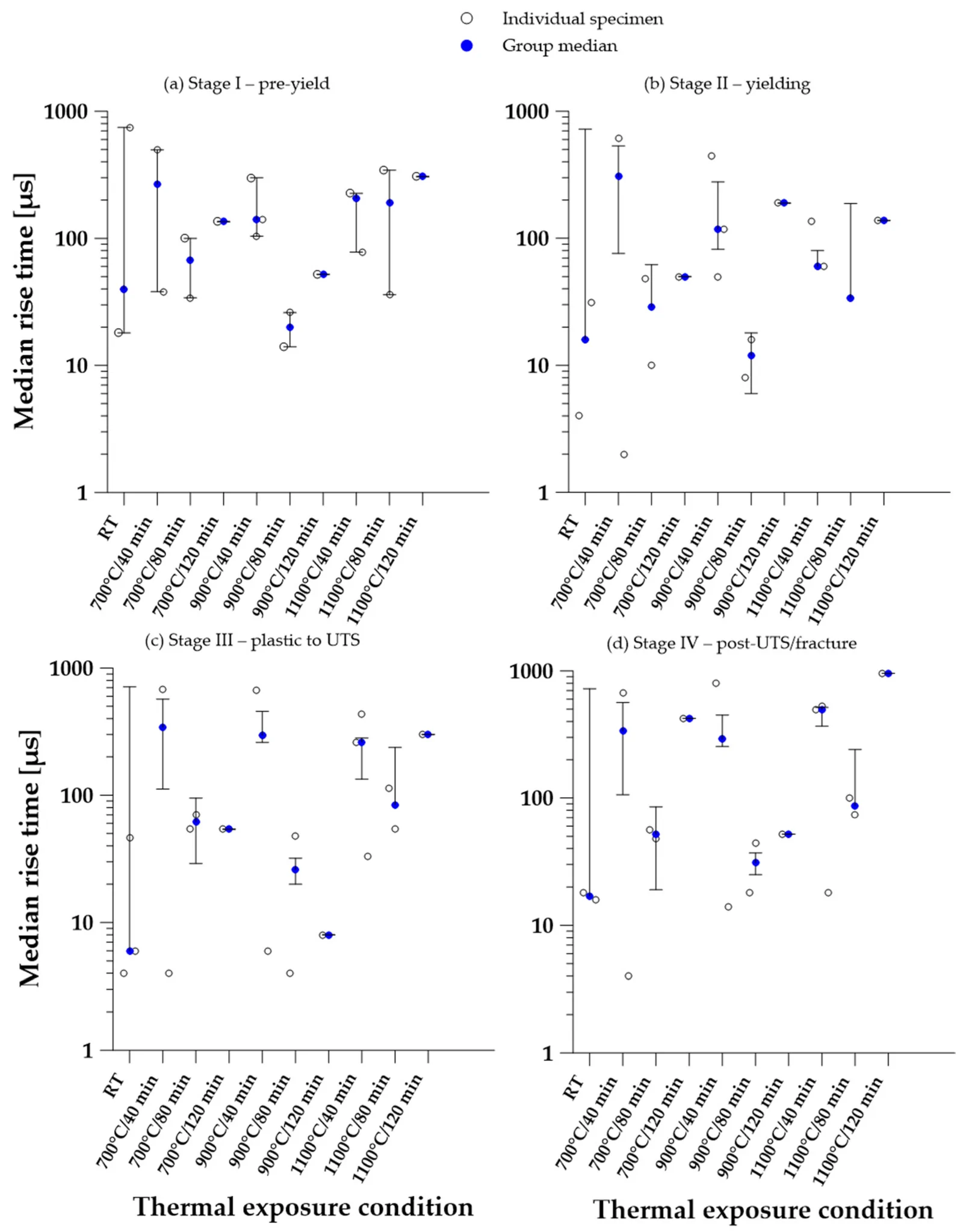
Specimen-level median rise time recorded by Channel 1 for the investigated nominal furnace-exposure conditions: (**a**) Stage I—pre-yield; (**b**) Stage II—yielding; (**c**) Stage III—plastic deformation to UTS; and (**d**) Stage IV—post-UTS localization and fracture. Rise time is presented on a logarithmic scale. Open symbols represent individual specimen medians, filled symbols represent condition medians, and vertical bars show the observed minimum–maximum range. Conditions represented by one available specimen are shown without a range and should be interpreted as exploratory observations.

The reference condition showed comparatively short median rise times after yielding, whereas several high-temperature conditions displayed longer rise times during plastic deformation or the post-UTS stage. Comparatively high Stage IV values were observed for selected high-temperature conditions, although the available specimen numbers were unbalanced. Rise time therefore revealed distinctions in the temporal development of the AE waveforms that were not reflected proportionally in their median amplitude. The stage-resolved rise-time results are presented in Figure 7.

### 3.8. Threshold, Initial-Loading, and Synchronization Sensitivity

The amplitude distribution was strongly influenced by the common 40 dB acquisition threshold. Of all 435,172 valid hits, 23.3% were recorded at exactly 40 dB, 35.5% did not exceed 41 dB, 46.8% did not exceed 42 dB, and 56.5% did not exceed 43 dB. More than half of the detected population was therefore located within 3 dB of the acquisition threshold.

The Stage I sensitivity analysis included 38 synchronized specimen–channel records. Excluding hits recorded before the mechanical stress reached either 5% or 10% of the specimen-specific UTS did not change any specimen-level Stage I amplitude median at the reported 1 dB resolution. For log_10_(absolute energy + 1), the changes ranged from −0.006 to 0.027 after applying the 5% UTS criterion and from −0.017 to 0.022 after applying the 10% UTS criterion. The median change across the evaluated records was 0.000 for both criteria. Thus, the reported Stage I medians were not driven solely by hits recorded during the earliest part of load introduction. Nevertheless, because source localization and a separate machine-noise reference test were not available, this analysis cannot establish that all retained Stage I hits originated within the specimen gauge section.

**Figure 8:**
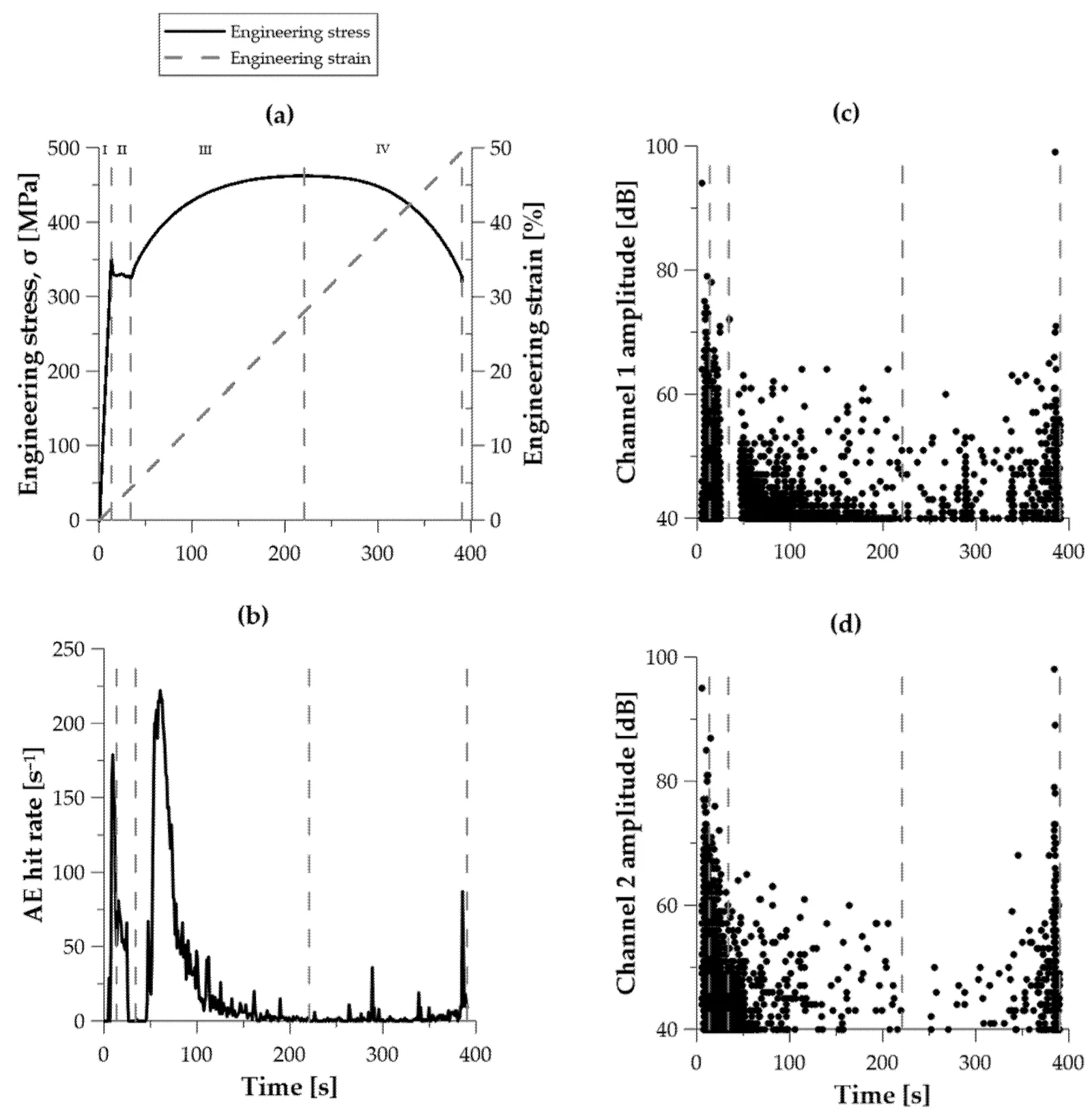
Representative common-time-axis presentation for specimen S235_0_1: (**a**) engineering stress and strain; (**b**) Channel 1 AE hit rate calculated in 1 s intervals; (**c**) amplitudes of individual Channel 1 hits; and (**d**) amplitudes of individual Channel 2 hits. Vertical dashed lines indicate *t*_YS_, *t*_YP,end_, *t*_UTS_, and *t*_f_. The figure illustrates the temporal relationship between the mechanical response and detected AE activity but does not provide source localization.

The stage assignments were also relatively insensitive to small changes in the synchronization offset. For boundary shifts of ±0.5 s, the median proportion of hits reassigned to another stage ranged from 0.74% to 0.90%, with a maximum of 1.87%. For shifts of ±1.0 s, the corresponding median proportion ranged from 1.57% to 1.91%, with a maximum of 3.55%. These results indicate that small synchronization changes affected only a limited fraction of the classified hits, although events occurring close to individual stage boundaries remain inherently sensitive to the selected offset. A representative common-time-axis presentation of the mechanical response and AE activity is provided in Figure 8.

## 4. Discussion

### 4.1. Stage-Dependent Evolution of AE Descriptors

The stage-resolved analysis indicates that the characteristics of the recorded AE hits changed during the transition from pre-yield deformation through yielding and plastic deformation to post-UTS localization. These changes were more clearly expressed by the temporal and energetic descriptors than by amplitude alone. Consequently, the AE response should be interpreted as a multidimensional signal evolution rather than as a uniform increase or decrease in acoustic activity.

Stage I extended from the beginning of the synchronized record to the upper yield point and therefore included load introduction as well as the subsequent pre-yield response. The comparatively elevated Stage I medians may reflect early material-related activity preceding macroscopic yielding, but contributions from specimen seating, friction, grip interaction, and measurement-chain stabilization cannot be excluded [29,38]. The sensitivity analysis showed that removal of hits recorded below 5% or 10% of the specimen-specific UTS did not change the Stage I amplitude medians and produced only minor changes in the transformed absolute-energy medians. This indicates that the reported specimen-level Stage I pattern was not determined solely by the earliest loading interval. However, the experiment did not include source localization, guard sensors, or a separately characterized machine-noise baseline. The elevated Stage I response therefore cannot be attributed exclusively to irreversible deformation within the gauge section.

The transition to Stage II and Stage III was generally accompanied by lower temporal and energetic medians. This evolution indicates that macroscopic yielding and subsequent distributed plastic deformation did not necessarily produce individual AE hits with progressively greater amplitude, duration, rise time, or absolute energy. In low-carbon structural steel, plastic deformation may proceed through numerous relatively weak acoustic events, while the measured characteristics of individual hits remain close to the acquisition threshold. Accordingly, a reduction in specimen-level medians does not imply an absence of AE activity or a reduction in the total number of recorded hits. Hit intensity and hit characteristics represent different aspects of the acoustic response and should not be treated interchangeably.

Changes in AE characteristics during the post-UTS period have previously been associated with the transition from distributed plastic deformation to localized necking and final fracture [19,20]. The accompanying modification of the propagation path and the concentration of deformation within a progressively smaller region may additionally influence the waveforms detected by each sensor [30]. The renewed increase in selected temporal and energetic descriptors observed here is compatible with this general transition, although the scalar hit parameters do not permit separation of the individual source and propagation effects.

### 4.2. Observations Across the Nominal Furnace-Exposure Conditions

Descriptive differences in AE characteristics were observed among the available records assigned to the nominal furnace temperature–duration groups, but they did not form a simple monotonic sequence with either furnace temperature or holding time. These observations should not be interpreted as statistically confirmed thermal-condition effects because the AE subset was incomplete and unbalanced, with some conditions represented by only one available specimen. In addition, the reported temperatures describe the furnace chamber rather than directly measured specimen temperatures. The present results therefore document differences among the investigated records under the specified nominal exposure protocol but do not establish a population-level causal relationship between a verified specimen thermal history and the subsequent AE response.

The conditions producing the lowest residual strength did not consistently produce the lowest or highest values of all AE parameters. Conversely, elevated temporal or energetic characteristics were observed for selected stages under several thermally exposed conditions without a corresponding proportional change in strength. This lack of a one-to-one relationship is reasonable because tensile properties and hit-level AE descriptors characterize different aspects of specimen behaviour. Mechanical parameters describe the macroscopic load-bearing response, whereas AE descriptors are also affected by the source process, source location, signal propagation path, sensor coupling, acquisition threshold, and temporal distribution of acoustic activity.

The results therefore support the use of stage-resolved comparisons rather than a single AE measure calculated for the complete tensile test. Combining all hits from different deformation stages could conceal changes occurring during yielding, distributed plastic deformation, or post-UTS localization. Mechanical referencing provides a physically interpretable basis for comparing signals recorded from specimens whose test durations and deformation histories differ.

The material was identified as S235 from the available documentation, but independent chemical-composition measurements, grade verification, and post-exposure microstructural observations were not available. Moreover, specimen-attached thermocouple measurements and complete specimen-specific heating and cooling curves were not recorded. Consequently, the observed mechanical and AE differences cannot be assigned to grain growth, phase transformations, recovery, recrystallization, oxidation, or other specific metallurgical mechanisms. Such interpretations require additional compositional, thermal, and microstructural evidence. The present findings should therefore be restricted to the investigated steel product and the documented nominal furnace-exposure protocol.

### 4.3. Complementarity of AE Descriptors

The influence of the detection threshold on AE amplitude distributions is well recognized because only signals exceeding the selected threshold are registered as hits [17,39]. A fixed threshold improves consistency between tests but also produces a truncated detected population and may concentrate observations near the lower acquisition boundary. In the present dataset, 23.3% of the valid hits were recorded at exactly 40 dB and 56.5% were within 3 dB of the threshold. Accordingly, the narrow amplitude range observed in the present results should be interpreted partly in relation to the common 40 dB acquisition setting.

The limited differentiation provided by amplitude does not mean that this descriptor is uninformative. Amplitude reflects the maximum voltage of a detected waveform and remains useful for identifying comparatively strong individual signals. However, two hits with similar peak amplitudes may have substantially different durations, rise times, waveform shapes, and integrated energies. Amplitude should therefore be interpreted together with temporal and energetic descriptors.

Duration and rise time provided complementary information concerning the temporal development of the detected signals. Their greater variation, particularly during post-UTS deformation, suggests sensitivity to changes that were not apparent from amplitude alone. Longer duration or rise time may result from a prolonged source process, repeated threshold crossings, waveform dispersion, reflections, or overlapping acoustic contributions [17,24,27]. These alternatives cannot be distinguished using the scalar hit descriptors alone, but they confirm that temporally extended signals are not equivalent to high-amplitude signals.

Absolute energy further complemented the amplitude-based assessment because it represents an integrated measure derived from the recorded waveform rather than its peak value alone. Energy-related AE descriptors can distinguish signals having comparable peak amplitudes but different durations and waveform contents, although the relationship among amplitude, duration, and energy is not unique [17,27]. In the present study, absolute energy values were obtained directly from the AE acquisition software and transformed as log_10_(*E*_abs_ + 1) because of their broad and right-skewed distribution. Although the values are reported in attojoules by the acquisition system, they remain dependent on sensor response, coupling, amplification, attenuation, propagation distance, and acquisition settings [28,29]. They should therefore be treated as configuration-dependent comparative descriptors rather than as direct measurements of the total mechanical energy released by the material.

Taken together, the descriptors indicate that the evolution of AE during tensile deformation cannot be represented adequately by a single scalar parameter. Amplitude describes signal magnitude, duration and rise time describe temporal characteristics, and absolute energy provides an integrated waveform-related measure. Their joint use offers a more complete description of stage-dependent AE behaviour, provided that conclusions remain tied to the specific measurement configuration.

### 4.4. Measurement-Channel Sensitivity

The paired specimen–stage analysis demonstrated that the two channels were not interchangeable. Amplitude showed the strongest correspondence, but even for this descriptor the concordance correlation coefficient was only 0.291 and the limits of agreement covered an interval of approximately 10 dB. Concordance was close to zero for duration and rise time, while log_10_(absolute energy + 1) also showed weak agreement.

These discrepancies are consistent with the measurement-chain dependence of scalar AE descriptors. The two sensors received signals through different propagation paths, and their records could be affected differently by source position, directivity, attenuation, reflections, coupling, and distance from the active deformation zone [29,30,40]. The results do not establish which of these factors produced the observed differences because coupling was checked before the tests using pencil-lead breaks but was not normalized quantitatively between all records.

Channel 1 was retained as the principal display channel to provide a consistent presentation without pooling signals detected through different measurement paths. Channel 2 was analyzed separately as a sensitivity assessment. Accordingly, differences in duration, rise time, and absolute energy among the nominal furnace-exposure groups should be understood as channel-specific observations rather than intrinsic material properties. The paired values for both channels are supplied in the Supplementary Materials.

### 4.5. Limitations and Implications

The principal limitation is the incomplete and unbalanced AE dataset. Although the mechanical programme comprised 30 specimens, archived AE records suitable for stage-resolved analysis were available for 20 specimens. The causes of the unavailable records were not documented sufficiently to determine whether their absence was random with respect to exposure condition or mechanical response. In addition, the 1100 °C/120 min group was represented by one complete AE record. The analysis is therefore exploratory and does not establish statistically confirmed differences among all nominal furnace-exposure conditions. The large number of individual hits cannot compensate for the limited number of independent specimens.

The thermal histories were also characterized incompletely. The reported temperatures were measured in the furnace chamber and no thermocouples were attached directly to the specimens. Complete heating and cooling curves, heating and cooling rates, time to specimen thermal equilibrium, furnace spatial-uniformity measurements, and removal-delay records were unavailable. The findings should therefore be interpreted in relation to nominal furnace-exposure groups rather than precisely verified specimen temperature histories.

Stage I included the introduction of load and could contain emissions associated with seating, grip interaction, friction, or stabilization of the measurement chain. Excluding hits recorded below 5% and 10% of specimen-specific UTS did not materially change the Stage I specimen medians, but the absence of source localization, guard sensors, and an independently measured machine-noise baseline prevents complete separation of material-related activity from test-system contributions.

Mechanical stage boundaries were determined individually from the corresponding stress–time and stress–strain records. The end of the yield plateau was selected visually as the onset of sustained strain hardening and therefore retained an element of operator judgement. Sensitivity analysis showed that shifts of ±0.5 s and ±1.0 s reassigned only a limited proportion of hits, but events occurring directly adjacent to a boundary remain sensitive to synchronization uncertainty.

The common 40 dB threshold restricted the analysis to the detected population and produced a pronounced concentration of hits close to the lower acquisition boundary. Signals below the threshold were not evaluated. Furthermore, the modest channel agreement demonstrates that the reported scalar descriptors depend on sensor position, propagation path, coupling, and the acquisition chain. Channel-specific values should not be transferred directly to other measurement configurations.

Finally, chemical-composition measurements, independent steel-grade verification, and microstructural characterization were not available. The observed differences cannot therefore be assigned to particular metallurgical transformations. Future studies should employ balanced specimen groups, directly measured specimen thermal histories, quantified coupling checks, source-localization or guard-sensor arrangements, and complementary microstructural observations. Within these limitations, specimen-specific mechanical segmentation remains useful for organizing AE records according to physically interpretable periods of tensile deformation.

## 5. Conclusions

This exploratory study examined stage-resolved scalar AE descriptors in the available records from tensile tests of an S235 steel product subjected to nominal furnace exposure. The principal conclusions are as follows:

1. In the available Channel 1 records, specimen-level median amplitude was generally highest during Stage I and lower during yielding and distributed plastic deformation. However, Stage I included load introduction, and the measurements did not permit complete separation of gauge-section emissions from possible grip- or machine-related contributions.
2. Duration, rise time, and transformed absolute energy exhibited greater inter-specimen and channel-dependent variability than amplitude. Selected records showed renewed increases in temporal or energetic descriptors during post-UTS deformation, but these observations cannot be assigned to individual source mechanisms using scalar hit parameters alone.
3. The two acquisition channels were not interchangeable. Agreement was modest for amplitude and weak for absolute energy, duration, and rise time, confirming that the reported values depend on the sensor position and measurement path.
4. Differences were observed among records representing the investigated nominal furnace temperature–duration groups, but the incomplete and unbalanced AE dataset does not support statistically confirmed condition-level thermal effects. The results should be treated as an exploratory specimen-referenced description specific to the investigated steel product and measurement configuration.

The study supports the use of specimen-specific mechanical segmentation for organizing AE data, while also demonstrating that independent specimen replication, verified thermal histories, source-discrimination procedures, and measurement-chain validation are required before stage-resolved AE descriptors can be used for generalized post-fire material assessment.

## Figures and Tables

**Table 1:** Residual mechanical properties of the investigated S235 steel product after nominal furnace exposure (mean ± SD; *n* = 3 mechanically tested specimens per condition). SD, standard deviation.

Condition	Lower Yield Strength [MPa]	Upper Yield Strength [MPa]	UTS [MPa]
RT	324.14 ± 2.40	351.01 ± 19.43	463.06 ± 1.27
700 °C/40 min	317.67 ± 4.27	349.19 ± 10.59	447.62 ± 1.76
700 °C/80 min	293.70 ± 5.33	315.65 ± 6.84	436.41 ± 7.71
700 °C/120 min	272.50 ± 6.03	319.22 ± 7.01	418.48 ± 6.70
900 °C/40 min	326.61 ± 2.87	338.88 ± 10.89	458.85 ± 0.80
900 °C/80 min	302.19 ± 6.56	311.45 ± 8.95	456.48 ± 3.90
900 °C/120 min	279.81 ± 8.55	294.00 ± 4.49	450.45 ± 6.73
1100 °C/40 min	266.18 ± 4.20	273.15 ± 2.79	425.09 ± 3.79
1100 °C/80 min	232.32 ± 5.84	—	390.14 ± 2.40
1100 °C/120 min	231.01 ± 11.51	240.53 ± 8.95	382.04 ± 10.14

**Table 2:** Paired specimen–stage comparison between Channels 1 and 2. Mean differences were calculated as Channel 1 minus Channel 2. CCC denotes Lin’s concordance correlation coefficient. The Bland–Altman limits of agreement were calculated as the mean paired difference ±1.96 standard deviations of the paired differences.

Descriptor	Paired Specimen–Stage Observations, n	Spearman ρ	Lin’s CCC	Mean Difference Ch1 − Ch2	Bland–Altman Limits of Agreement
Amplitude [dB]	78	0.434	0.291	−1.673	−6.670 to 3.324
log_10_(absolute energy + 1)	78	0.198	0.099	0.402	−0.696 to 1.501
Duration [µs]	78	0.076	−0.002	528.9	−731.8 to 1789.7
Rise time [µs]	78	0.011	−0.002	165.3	−276.3 to 606.8

## Data Availability

The original contributions presented in this study are included in the article/Supplementary Materials. Further inquiries can be directed to the author.

## Supplementary Materials

The following supporting information can be downloaded at: https://www.mdpi.com/article/10.3390/ma19214495/s1, Table S1: Accounting of all mechanically tested specimens and availability of stage-resolved AE records, including retained stage-boundary times and durations; Table S2: Number of AE hits by analyzed specimen, acquisition channel, and deformation stage; Table S3: Paired condition- and stage-resolved AE descriptor medians for Channels 1 and 2; Table S4: Specimen-, channel-, and stage-resolved AE descriptor medians, interquartile ranges, hit counts, and threshold-proximity fractions; Table S5: Paired agreement analysis between Channels 1 and 2; Table S6: Sensitivity of Stage I medians to exclusion of the initial low-load interval; Dataset S1: Underlying specimen-level medians; Dataset S2: Sensitivity of stage assignment to artificial time shifts.
